# New Insights into the Potential Cytotoxic Role of *Bacillus cytotoxicus* Cytotoxin K-1

**DOI:** 10.3390/toxins13100698

**Published:** 2021-10-01

**Authors:** Klèma Marcel Koné, Pauline Hinnekens, Jelena Jovanovic, Andreja Rajkovic, Jacques Mahillon

**Affiliations:** 1 Laboratory of Food and Environmental Microbiology, Université Catholique de Louvain (UCLouvain), 1348 Louvain, Belgium; kklema3@gmail.com (K.M.K.); pauline.hinnekens@uclouvain.be (P.H.); 2 Department of Food Technology, Safety and Health, Research Group of Food Microbiology and Food Preservation, Faculty of Bioscience Engineering, Ghent University (UGent), 9000 Ghent, Belgium; Jelena.Jovanovic@UGent.be (J.J.); Andreja.Rajkovic@UGent.be (A.R.)

**Keywords:** *Bacillus cereus*, *Bacillus cytotoxicus*, CytK-1, cytotoxicity, Caco-2

## Abstract

The thermotolerant representative of the *Bacillus cereus* group, *Bacillus cytotoxicus*, reliably harbors the coding gene of cytotoxin K-1 (CytK-1). This protein is a highly cytotoxic variant of CytK toxin, initially recovered from a diarrheal foodborne outbreak that caused the death of three people. In recent years, the cytotoxicity of *B. cytotoxicus* has become controversial, with some strains displaying a high cytotoxicity while others show no cytotoxicity towards cell lines. In order to better circumscribe the potential pathogenic role of CytK-1, knockout (KO) mutants were constructed in two *B. cytotoxicus* strains, E8.1 and E28.3. The complementation of the *cytK-1* KO mutation was implemented in a mutant strain lacking in the *cytK-1* gene. Using the tetrazolium salt (MTT) method, cytotoxicity tests of the *cytK-1* KO and complemented mutants, as well as those of their wild-type strains, were carried out on Caco-2 cells. The results showed that *cytK-1* KO mutants were significantly less cytotoxic than the parental wild-type strains. However, the complemented mutant was as cytotoxic as the wild-type, suggesting that CytK-1 is the major cytotoxicity factor in *B. cytotoxicus.*

## 1. Introduction

Cytotoxin K-1 (CytK-1) is a highly cytotoxic and necrotic variant of cytotoxin K (CytK). It was initially recovered from a specific *Bacillus cereus* strain isolated from a food poisoning outbreak fatal to three elderly people in France in 1998. This *B. cereus* strain and its kin were later described as a new species, *Bacillus cytotoxicus*, the thermotolerant representative of the *B. cereus* group [1,2]. This group, also known as *B. cereus sensu lato* (*s.l.*), contains Gram-positive, spore-forming, and facultative anaerobic bacteria. Although numerous new species have been described as *B. cereus s.l.* members, the primary members of the group are *B. cereus sensu stricto (s.s.)*, *Bacillus thuringiensis*, *Bacillus anthracis*, *Bacillus mycoides*, *Bacillus pseudomycoides*, and *Bacillus weihensphanensis*. The *B. cereus* group comprises both beneficial and pathogenic members. While *B. mycoides*, *B. pseudomycoides,* and *B. weihensphanensis* have not reportedly been implicated in any human infections or foodborne diseases yet [3], many *B. thuringiensis* have been used for several decades as bio-pesticides in agriculture and control of disease vectors, due to their ability to produce insecticidal molecules [4].

Because of its plasmid-encoded anthrax toxins, *B. anthracis* is highly pathogenic to mammals, including humans [5], and some *B. cereus s.s.* strains have been implicated in human extra-gastrointestinal infections, as well as in foodborne illnesses [6]. Indeed, an ingestion of food containing particular *B. cereus* strains can lead to two types of foodborne diseases, the emetic and diarrheal syndromes. The former is caused by a plasmid-encoded, in-food produced, heat-stable, acid-resistant and ring shape 1.2 kDa peptide, the cereulide [7,8]. The diarrheal syndrome is presumably caused by one or a combination of several enterotoxins produced in the small intestine after ingestion of *B. cereus* contaminated food. These enterotoxins are chromosomally encoded and prominently include the hemolysin BL (Hbl), the non-hemolytic enterotoxin (Nhe) and CytK [9]. The expression of most of these potential enterotoxins is regulated by the Pap/PlcR regulatory system [10]. Hbl and Nhe toxins are both three-component toxins. While almost all *B. cereus s.l.* members harbor the Nhe genes (*nheABC*), about 40 to 60% contain the Hbl genes (*hblABCD)* [11,12]. It has also been shown that the Nhe toxin initiate cell apoptosis in Vero cells [13], and more recently, it was reported that Hbl and Nhe can act synergistically to trigger inflammation [14].

Encoded by the *cytK* gene, CytK is a 34-kDa single-peptide toxin for which two variants have been reported, CytK-1 and CytK-2. The latter forms smaller pores in phospholipidic bilayer membrane, is less cytotoxic, and is mainly found in some mesophilic *B. cereus* strains. It shares 87% amino acid identities with CytK-1 toxin which is more cytotoxic, and whose coding gene is restricted to *B. cytotoxicus* strains [15]. However, NVH 883-00, a *B. cytotoxicus* strain originating from spices, was shown to under-express CytK-1 toxin and be less cytotoxic than the reference strain, NVH 391-98, isolated from the fatal outbreak in France [16]. Moreover, based on whole genome sequencing, four genomic clades (A to D) have been described and clade A reportedly gathered the most potentially cytotoxic strains, including strains NVH 391-98 and CH_213 [17,18]. It is also noteworthy that the presence of toxin genes does not by itself lead to their expression and to the bacterium cytotoxicity. Indeed, the expression of *B. cereus* enterotoxin genes is thoroughly regulated and is dependent on the strain and its microenvironment [19,20,21]. It has been suggested that the cytotoxicity of *B. cytotoxicus* could have been overestimated in the past. As for other potentially enterotoxigenic *B. cereus*, the cytotoxicity of *B. cytotoxicus* should not be deduced solely based on the presence/absence of the *cytK-1* gene and genetic profile should thus be complemented with (cyto)toxicity assays [20,22].

Therefore, there was a need for an in-depth exploration of the implication of CytK-1 toxin in *B. cytotoxicus* toxicity. To the best of our knowledge, no study has yet focused on the deletion of *cytK-1* gene in order to assess the role of its toxin. Hence, the current study aimed at constructing *B. cytotoxicus* mutants lacking their *cytK-1* gene and comparing their cytotoxicity with that of the parental wild-type strains.

## 2. Results and Discussion

### 2.1. Construction of E28.3^cytK-1 KO-A^ and E8.1^cytK-1 KO^ B. cytotoxicus Mutants Lacking cytK-1

Since the initial isolation of *B. cytotoxicus* from a fatal food poisoning incident [1], its toxicity has become somewhat controversial. Indeed, it has even been suggested that the cytotoxicity of this species was overestimated in the past [20]. To help circumscribe the role of CytK-1 toxin, we knocked out the *cytK-1* gene in two *B. cytotoxicus* strains, E28.3 and E8.1. Both strains were isolated from potato flakes. In contrast to the reference strain NVH 391-98 showing no visible plasmid, E28.3 and E8.1 display large and small plasmids. These two *B. cytotoxicus* strains share the same RAPD profile, which is different from that of NVH 391-98 [23]. They also display distinct plasmid profiles. While strain E28.3 is one of the rare *B. cytotoxicus* of the collection to be susceptible to electroporation, E8.1 is the only strain in which the *cytK-1* KO mutation could be successfully transferred via conjugation (see below).

**Figure 1:**
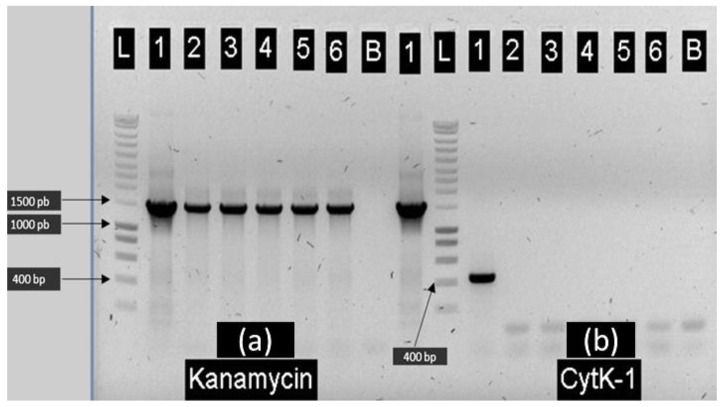
PCR-based confirmation of the five *B. cytotoxicus* KO-mutants selected after homologous recombination. L: DNA molecular weight markers (200 bp to 10 kb); B: negative control (buffer only). Panel (**a**): PCR-screening of the Kanamycin resistance gene: lanes 1 to 6: positive control (pDG173), E28.3*^cytK−1^*^-KO-A^, E28.3*^cytK−1^*^-KO-B^, E28.3*^cytK−1^*^-KO-C^, E28.3*^cytK−1^*^-KO-D^ and E28.3*^cytK−1^*^-KO-E^. Panel (**b**): Detection of the *cytK-1* gene: lanes 1 to 6: positive control (E28.3 wild-type), E28.3*^cytK−1^*^-KO-A^, E28.3*^cytK−1^*^-KO-B^, E28.3*^cytK−1^*^-KO-C^, E28.3*^cytK−1^*^-KO-D^ and E28.3*^cytK−1^*^-KO-E^.

Using a double-recombination approach based on a thermo-sensitive shuttle plasmid, a KO-mutant of the *cytK-1* gene was built in strain E28.3 by replacing this locus by a kanamycin-resistance (*kan^R^*) gene. Five recombinant strains (A–E) were screened for *cytK-1* and kanamycin resistance genes. As shown in Figure 1, all the potential recombinant clones carried the resistance gene (Lanes a2 to a6), while they all lost the *cytk-1* gene (Lanes b2 to b6). Additional PCR experiments confirmed the validity of the gene swap (data not shown). Based on these results, the E28.3*^cytK-1-^*^KO-A^ recombinant clone was retained for further experiments.

**Figure 2:**
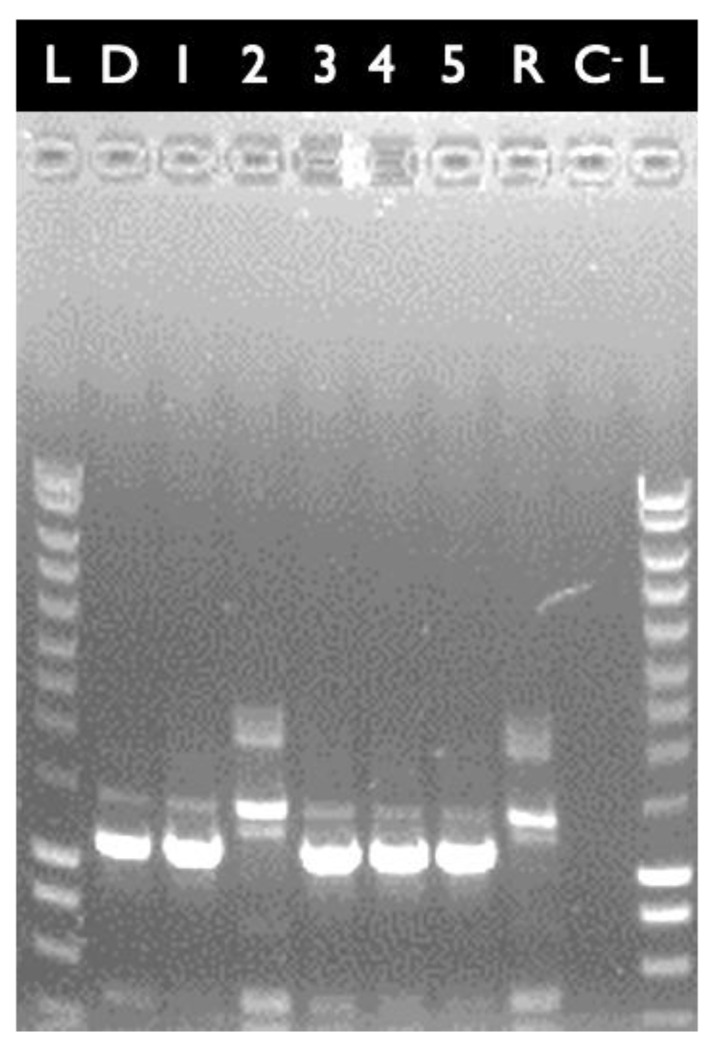
RAPD pattern of *B. cytotoxicus* strains using the OPA9 primer. L: DNA molecular weight markers (200 bp to 10 kb). D: donor strain E28.3*^cytK−1^*^-KO-A^; R: recipient strain E8.1; C^-^: negative control (buffer only); Lanes 1 to 5: candidate transconjugants. Lane #2 contains a *bona fide* E8.1 transconjugant containing the desired genetic loci (i.e., *kan^R^* gene replacing the *cytK-1* gene). It was named E8.1*^cytK−1-^*^KO-B^.

It was recently shown that the mega-plasmid pXO16 could mobilize chromosomal loci between members of the *B. cereus* group during its conjugation [24]. Using this approach, the KO-mutation of E28.3*^cytK−1-^*^KO-A^ was transferred to strain E8.1 via filter mating conjugation. The kanamycin-resistant (Kan^R^) candidate transconjugants were then verified by RAPD profiling [23] to confirm that they displayed the same pattern as their parental strain E8.1. As shown in Figure 2, the clone present in Lane 2 displayed the RAPD pattern identical to that of its parental strains E8.1. Additional PCR confirmed that this clone (named E8.1*^cytK−1-^*^KO-B^) had acquired the locus where *cytK-1* was replaced by the *Kan^R^* gene (data not shown). This clone was retained for the cytotoxicity experiments.

Given that viable mutants of *B. cytotoxicus* strains lacking the *cytK-1* gene were obtained, it can be suggested that this gene is not essential to *B. cytotoxicus*. However, it is worth mentioning that attempts to create a similar mutant in the reference type-strain NVH 391-98 through electroporation, as well as via pXO16 conjugation, have failed (data not shown). The reasons for these unsuccessful attempts remain so far unknown. Future trials to construct mutants of other highly cytotoxic *B. cytotoxicus* strains, such as CH_213 [18], should certainly be considered.

### 2.2. Cytotoxicity of B. cytotoxicus Wild-Type Strains E28.3 and E8.1, Their Derived Mutants Lacking the cytK-1 Gene and the Complemented Mutant

**Figure 3:**
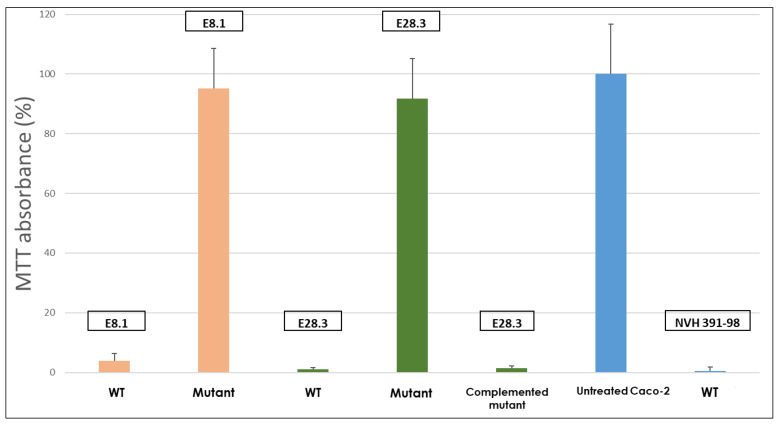
Tetrazolium salt method (MTT) used to assess viability of Caco-2 cells after 12 h of exposure to *B. cytotoxicus* supernatants. Cells treated with supernatant of wild-type (WT) NVH 391-98 and untreated Caco-2 cells were used as positive and negative control, respectively. Mutants (E8.1*^cytK−1-^*^KO^ and E28.3*^cytK−1^*^-KO-A^) lacking the *cytK-1* gene are less cytotoxic than the WT strains of E8.1 and E28.3 (*p* < 0.05). The complemented mutant of E28.3*^cytK−1^*^-KO-A^ is as cytotoxic as the WT E28.3.

Caco-2 cells were exposed to the *B. cytotoxicus* supernatant of the reference strain NVH 391-98, as well as that of wild-type strains E8.1 and E28.3 and that of the derived *cytK-1* KO-mutants. The cytotoxicity was assayed using the tetrazolium salt method (MTT) which assesses cell viability. After two hours, the highly cytotoxic strain, NVH 391-98, was able to impair Caco-2 viability while little cytotoxicity effect was observed for the wild-type strains E8.1 and E28.3. However, after twelve hours of exposure, these two strains were almost as cytotoxic as NVH 391-98 (Figure 3).

Interestingly, deleting the *cytK-1* gene had a striking effect: in both genetic backgrounds, removing the gene reduced by more than 90% the deleterious effect of the E28.3*^cytK−1^*^-KO-A^ and E8.1*^cytK−1-^*^KO-B^ supernatants on Caco-2 cell viability, indicating a drastic reduction of cytotoxicity and suggesting that CytK-1 toxin plays a major role in *B. cytotoxicus* cytotoxicity. In order to confirm that the observed effects were directly related to the *cytK-1* gene deletion, a complementation experiment was conducted by cloning *cytK-1* into the pHT304-18Z shuttle vector and introducing it in the E28.3*^cytK−1^*^-KO-A^ mutant by electroporation. As shown in Figure 3, the resulting complemented mutant displayed almost the same activity as its wild-type counterpart, reinforcing the idea that CytK-1 is responsible for most of the *B. cytotoxicus* cytotoxicity, at least on Caco2 cells.

The toxicity of NVH 391-98, initially isolated from the fatal case in 1998, is in line with previous reports [1,20]. Recently, Stevens et al. [17] explored the relationship between genomic diversity and cytotoxicity among strains pertaining to four *B. cytotoxicus* genomic clades (A–D). It was then suggested that potentially highly cytotoxic strains are gathered in clades A and B, while those of clades C and D are presumably less cytotoxic. More recently, the draft genome of another highly cytotoxic *B. cytotoxicus* strain, CH_213, pertaining to genomic clade A has been released [18]. In addition, whole genome sequencing of wild-type strains E8.1 and E28.3 indicated that they relate to clade C [25]. With clade C strains displaying relatively high cytotoxicity toward cell lines, the current findings indicate that highly cytotoxic *B. cytotoxicus* strains are not necessarily restricted to clades A and B. Nevertheless, a better understanding of the link between genomic grouping and virulence would require cytotoxic studies on other *B. cytotoxicus* strains, from different food matrices and genomic clades.

The inactivation of *cytK-1* correlates with a drastic drop in the cytotoxicity of our *B. cytotoxicus* strains. This is in contrast to the findings of Romarao and Lereclus, in which there was no significant cytotoxicity reduction (both on Caco-2 and HeLa cell lines) in a *B. thuringiensis* mutant lacking its *cytK-2* gene [26]. It is noteworthy that the *cytK-2* gene harbored by *B. thuringiensis* encodes for the less cytotoxic variant of the CytK toxin [15]. Assessing the various wild-type *B. cytotoxicus* and *B. thuringiensis* strains and their *cytK*-KO mutants in the same assay could be valuable to resolve this seeming discrepancy.

Despite displaying an impaired cytotoxicity, our KO-mutants potentially possessed remaining toxicity (Figure 3) that could be imputable to other to-be-investigated enterotoxins or virulence factors in *B. cytotoxicus*. In fact, previous reports have shown that *B. cytotoxicus* lacks the *Hbl* genes but harbors a novel variant of *Nhe* genes [1,16]. Nhe is a three-component toxin that was shown to be necrotic at high concentrations, while inducing apoptosis in sub-necrotic concentrations. It has also been recently reported to trigger inflammation synergistically with the Hbl toxin [13,14]. As for several *B. cereus* enterotoxins, Nhe and CytK-1 are under the control of the PlcR regulator [10], and it has been reported that NVH 391-98 overproduces the CytK-1 toxin [16]. We can therefore speculate that the high cytotoxicity of some *B. cytotoxicus* strains could be imputable to a synergistic action of large amount of produced CytK-1 and/or Nhe toxins. It remains to be seen whether the remaining toxicity displayed by the *cytK-1* KO-mutants can be attributed to these other putative enterotoxins.

## 3. Conclusions

In conclusion, viable *B. cytotoxicus* mutants lacking the *cytK-1* gene were successfully created. Cytotoxicity tests showed that these mutants were less cytotoxic than the parental wild-type strains. To give further credence to these observations, a complementation of the knockout mutants with the wild-type *cytK-1* gene was constructed. The complemented mutant displayed cytotoxic activity comparable to that of the wild-type strains. Together, these results suggest that CytK-1 toxin is indubitably implicated in *B. cytotoxicus* cytotoxicity. The exact contribution of this activity to the diarrheal syndrome caused by the *B. cytotoxicus* strains remains, however, to be clarified. Similarly, it is plausible that high cytotoxicity observed in certain *B. cytotoxicus* strains could be imputable to the association of CytK-1 with one or more additional enterotoxin(s) and/or enzyme(s).

## 4. Materials and Methods

### 4.1. Bacterial Strains, Growth Media and Plasmids

The bacterial strains and plasmids used in this study are summarized in Table 1. *B. cytotoxicus* strain E28.3 was used to create the *cytK-1* KO mutant through the homologous recombination method (see below). NEBuilder HiFi DNA Assembly Cloning Kit (New England *Biolabs*™, Ipswich, MA, USA) was used to insert the PCR amplicons into the thermo-sensitive plasmid pMAD [27]. Chemically competent *Escherichia coli* NEB 5-alpha and *dam^−^/dcm^−^* C2925 strains (New England *Biolabs*™, Ipswich, MA, USA) were used for the cloning steps. Plasmid pXO16 conjugation was used to transfer, via mobilization, the *cytK-1*-KO mutation into strain E8.1. Bacterial strains were grown at 37 °C (*E. coli*) or 30 °C (*B. cytotoxicus*) on Luria-Bertani (LB) medium (broth or supplemented with 1.5% agar) containing the appropriated antibiotics: ampicillin (Ap, 100 μg/mL), erythromycin (Ery, 10 μg/mL), kanamycin (Kan, 50 μg/mL) or tetracycline (Tet, 50 μg/mL).

### 4.2. Construction of the E28.3 Mutant Strain Lacking the cytK-1 Gene

A fresh overnight colony of *B. cytotoxicus* strain E28.3 from LB agar was mixed in 50 μL of deionized water for the DNA extraction. Using Q5 High-Fidelity polymerase (Promega, Leiden, The Netherlands), PCR amplification of upstream (987 bp) and downstream (1001 bp) regions of *cytK-1* gene, as well the kanamycin resistance gene from plasmid pDG783 [28], were performed. The primers used in this study are listed in Table 2. Electrophoresis gel migration of the PCR products was performed as described elsewhere [23]. The PCR amplicons were purified using GenElute™ PCR Clean-Up Kit (Sigma-Aldrich™, Overijse, Belgium) and the DNA quality was check with the Nanodrop spectrophotometer (Isogen, De Meern, The Netherlands).

**Table 1:** Bacterial strains and plasmids used.

Strains	Main Features	References
*B. cytotoxicus* strains		
E8.1	Wild-type strain isolated from potato flakes; RAPD pattern A, plasmid profile PP10	[23]
E8.1*^cytK−1^*^-KO-B^	*cytK-1* knockout mutant of E8.1	This work
E28.3	Wild-type strain isolated from potato flakes; RAPD pattern A and plasmid profile PP8	[23]
E28.3*^cytK−1^*^-KO-A^	*cytK-1* knockout mutant of E28.3	This work
E28.3.1	Derivative of E28.3 containing pXO16::Tn*5401*, Tet^R^	[29]
E28.3.1*^cytK−1^*^-KO-A^	Derivative of E28.3*^cytK−1^*^-KO-A^ containing pXO16::Tn*5401*, Tet^R^	This work
E28.3*^cytK−1^*^-KO-A^(pHT304-18Z::*cytK-1*)	Complementation of the mutant lacking *cytK-1* gene	This work
NVH 391-98	Reference strain; highly cytotoxic	[1]
***E. coli*** **strains**		
*E. coli* C2925 *dam^−^/dcm^−^*	K12 derivative deficient in adenine and cytosine methyl-transferases, chemically competent	New England *Biolabs*™
*E. coli* NEB 5-alpha	DH5-alpha derivative, T1 phage resistant and *endA1* deficient, chemically competent	New England *Biolabs*™
**Plasmids**		
pHT304-18Z	Shuttle vector used in the complementation; Ery^R^	[30]
pHT304-18Z::*cytK-1*	*CytK-1* gene of strain E28.3 cloned into the pHT304-18Z shuttle vector	This work
pMAD	Gram+/Gram- shuttle vector containing a thermo-sensitive Gram+ replicon; Ery^R^, Amp^R^	[27]
pMAD::UD	pMAD derivative containing the Kan^R^ cassette with up- and down-stream regions of the *cytK-1* gene, Ery^R^, Amp^R^, Kan^R^	This work
pDG783	Plasmid carrying a Kan^R^ cassette; Kan^R^	[28]
pXO16::Tn*5401*	Large conjugative plasmid from *B. thuringiensis* sv. *israelensis*, tagged with a Tetracycline-resistance gene; Tet^R^	[31]

As previously described by Gibson et al. [32], the purified PCR amplicons were mixed with the PCR-opened pMAD shuttle vector [27], deionized water, and NEBuilder HiFi DNA Assembly MasterMix. The HiFi DNA assembly reaction product was chemically transformed into *E. coli* NEB 5-alpha and incubated at 37 °C (1 h, 180 rpm). The bacterial culture was then spread on a LB agar supplemented with ampicillin and incubated overnight at 37 °C. Next, *E. coli* transformants were PCR-checked for the presence of the expected construct. The recombinant plasmid, pMAD::UD (containing the Kan^R^ cassette flanked by the upstream and downstream sequences of *cytK-1*) was extracted using GenElute™ Plasmid Miniprep Kit (Sigma-Aldrich™, Overijse, Belgium) and checked through DNA sequencing (Macrogen Europe, Amsterdam, The Netherlands). Thereafter, the construct was demethylated in *E. coli* C2925 before subsequent electroporation into *B. cytotoxicus*.

**Table 2:** Primers used in the cloning steps.

Designation	Primers	Sequences (5′ to 3′)	Tm (°C)	Amplicon (pb)	Reference
Kan^R^ gene	Kana_R	GTTTTTTACTATCGATACAAATTCCTCGTAG	60	1.490	This study
	Kana_F	ACATATATCGTGATAAACCCAGCGAACC			
*CytK-1* upstream region	cytK-1_Up_R	GGGTTTATCACGATATATGTCGTATTTCACATATATC	58	987	This study
	cytK-1_Up_F	AGATCTATCGATGCATGCCAGAAGTTTTAGGTTCATACATTTG			
*CytK-1* downstream region	cytK-1_Down_R	CGGATCCATATGACGTCGACAATGCGAGAGACGTTGCG	62	1.001	This study
	cytK-1_Down_F	TTGTATCGATAGTAAAAAACAACACTGACAAACTC			
*CytK-1* gene	cytK-1_R	CCTCGTGCATCTGTTTCATGAG	61	436	[33]
	cytK-1_F	CAATTCCAGGGGCAAGTGTC			

The *B. cereus* electroporation protocols previously described [34,35] were adapted for *B. cytotoxicus*. Briefly, a single fresh colony of E28.3 was sub-cultured in 25 mL of brain-heart infusion (BHI) (Bio-Rad, Richmond, CA, USA) and incubated O/N (30 °C, 120 rpm). Next, the bacterial culture was centrifuged (6000 rpm, 4 °C, 10 min), the cell pellet washed three times with chilled deionized water (4 °C) and resuspended in 400 μL PEG6000 (40% *wt*/*v*). Using the Gene Pulser and in a 2 mm cuvette (Bio-Rad, Richmond, CA, USA), the electro-competent *B. cytotoxicus* cells were electroporated with 1 to 2 μg of demethylated pMAD::UD plasmid. Electroporated bacterial cells were immediately suspended in 1 mL of LB broth and incubated at 30 °C, (1 h, 120 rpm). Next, 100 μL of the bacterial suspension were spread on LB agar supplemented with erythromycin and incubated O/N at 30 °C. The transformants were PCR-checked for the construct presence.

Swapping of the *cytK-1* gene by the *kan^R^* gene in *B. cytotoxicus* strain E28.3 was performed following the plasmid homologous recombination approach described by Makart et al. [24,36]. *B. cytotoxicus* transformants containing the shuttle pMAD::UD underwent successive non-permissive incubation cycles as follow: two at 43 °C, one at 45 °C and a last one at 50 °C. Up to 10^−7^ serial dilutions were made from the last cycle culture (50°C) and spread on LB agar supplemented with kanamycin and 20 μg/μL of X-Gal (Sigma-Aldrich™, Overijse, Belgium). Since pMAD contains a β-galactosidase cassette, five white colonies (indicating pMAD absence) on X-Gal LB agar were re-streaked on LB agar supplemented with kanamycin and incubated overnight at 30 °C.

### 4.3. Transfer of the cytK-1 KO Locus from Strain E28.3 to Strain E8.1

Using filter mating as described by Hinnekens et al. [29], the *cytK-1* KO mutation was mobilized from *B. cytotoxicus* strain E28.3.1*^cytK−1^*^-KO-A^ (Derivative of E28.3*^cytK−1^*^-KO-A^ containing pXO16::Tn*5401*) into E8.1 via conjugation [24]. The candidate transconjugants strains were PCR-screened to check the effective replacement of *cytK-1* gene by *Kan^R^* gene. RAPD patterns of the final transconjugant were also checked as previously described by Koné et al. [23].

### 4.4. Complementation of the cytK-1 KO Mutant

Primers flanked with overlapping restriction sites sequences of *Bam*H1 and *Pst*1 enzymes (New England *Biolabs*™, Ipswich, MA, USA) (Table 2) were used to amplify the *cytK-1* gene from *B. cytotoxicus* wild-type strain E28.3. As described by Makart et al. [24], the PCR product was cloned in the shuttle pHT304-18Z and transformed into chemically competent *E. coli* 10-beta cells. The resulting construct pHT304-18Z::*cytK-1* was retrieved using the GeneElute Plasmid Miniprep Kit (Sigma-Aldrich™, Overijse, Belgium) and checked through sequencing (Macrogen Europe, Amsterdam, The Netherlands). The pHT304::18Z-*cytK-1* construct was then demethylated by passing it through *E. coli* C2925 and subsequently electroporated into the *B. cytotoxicus* strain *cytK-1*-KO mutant to obtain E28.3*^cytK−1^*^-KO-A^(pHT304-18Z::*cytK-1*).

### 4.5. Cytotoxicity of E28.3, E8.1, Their Mutants Lacking cytK-1 and the Complemented Strain

#### 4.5.1. Cell Culture

The Caco-2 cell culture described by Rajkovic et al. [37] was adapted as follows. The human colorectal carcinoma cell line Caco-2 (HTB-37™) was obtained from American Type Culture Collection (ATCC) (Manassas, VA, USA). Cells were maintained in high glucose, Glutamax Dulbecco’s Modified Eagle Medium (DMEM) (Gibco-Thermo Fisher, Geel, Belgium), supplemented with (i) 10% heat-inactivated foetal bovine serum (Greiner Bio One, Wemmel, Belgium), (ii) 1% non-essential amino acids (Gibco-Thermo Fisher, Geel, Belgium), (iii) 1% penicillin/streptomycin (Gibco-Thermo Fisher, Geel, Belgium) in the humidified 10% CO_2_ atmosphere at 37 °C (Memmert GmbH & Co., Nurnberg, Germany). Cell morphology was regularly checked with phase-contrast microscopy (VWR, Leuven, Belgium) and the growth medium was changed every other day. After reaching 80–90% of confluency, the cells were sub-cultured by trypsinization using 0.5% trypsin-EDTA (Gibco-Thermo Fisher, Geel, Belgium) until cells were realized from the bottom of 75 cm^2^ neck tissue culture flasks (SPL Life Sciences, Gyeonggi-do, Korea). The cells were counted with a Bürker counting chamber according to the Trypan blue staining method. Next, they were seeded in a 96-well plate at a concentration of 20,000 cells per well to achieve optimal distribution of cells. In all experiments, the cells were passaged less than 25 times.

#### 4.5.2. Preparation of Bacterial Cell-Free Supernatants

To assess the effects of the wild-type *B. cytotoxicus* strains E28.3, E8.1, their mutant *cytK-1-KO* derivatives and the complemented strain E28.3*^cytK−1^*^-KO-A^(pHT304-18Z::*cytK-1*), cell-free supernatants were prepared. *B. cytotoxicus* reference strain NVH 391-98 cell-free supernatant and the untreated Caco-2 cells were used as positive and negative controls, respectively. Single separated colonies of selected strains were inoculated in serum and antibiotic-free DMEM and incubated for overnight at 37 °C. The cultures were centrifuged at 5000 *g* for 10 min, and supernatants were filter-sterilized through a 0.22 um filter (Millipore Inc., Billerica, MA, USA) and used freshly. Two-fold diluted supernatants were used for Caco-2 cell exposure, to better discriminate cytotoxicity of the strains. Exposure was performed for 2, 4, and 12 h. The effects were the most notable after 12 h of exposure.

#### 4.5.3. MTT Assay

Cytotoxic effects were characterized through the tetrazolium salt or MTT [(3-(4,5-dimethylthiazol-2-yl)-2,5-diphenyltetrazolium bromide]. This method assesses the cell’s mitochondrial activity as an indicator of cell viability and cytotoxicity, initially described by Mosmann [38]. After exposure, 20 μL of MTT (5 mg/mL in PBS) was added to each well, and the plates were incubated at 37 °C for 2 h. Upon incubation, liquid was discarded, and the purple formazan crystals were solubilized in dimethyl-sulfoxide (DMSO). SpectraMax plate reader (Molecular Devices, Sunnyvale, CA, USA) was used to record the absorbance at 570 nm.

#### 4.5.4. Statistical Analysis

The normality of the data was investigated using the Kolmogorov–Smirnov test. Microsoft Excel 2016 was used to compute mean values and standard deviations (N = 2, n = 6) for each test condition. To determine whether the data were significantly different (*p* < 0.05), a *t*-test (two-tailed with unequal variance) was performed with SPSS Statistics 26 (Chicago, IL, USA).
